# The Role of Charge Transfer in the Formation of Type I Deep Eutectic Solvent-Analogous Ionic Liquid Mixtures

**DOI:** 10.3390/molecules24203687

**Published:** 2019-10-14

**Authors:** Dinis O. Abranches, Nicolas Schaeffer, Liliana P. Silva, Mónia A. R. Martins, Simão P. Pinho, João A. P. Coutinho

**Affiliations:** 1 CICECO-Aveiro Institute of Materials, Department of Chemistry, University of Aveiro, 3810-193 Aveiro, Portugal; 2 Associate Laboratory LSRE-LCM, Instituto Politécnico de Bragança, Campus de Santa Apolónia, 5300-253 Bragança, Portugal; 3 Centro de Investigação de Montanha (CIMO), Instituto Politécnico de Bragança, Campus de Santa Apolónia, 5300-253 Bragança, Portugal

**Keywords:** charge transfer, quaternary ammonium, ionic liquid mixtures, deep eutectic solvents, solid–liquid equilibria, molecular dynamics

## Abstract

It was recently shown that tetramethylammonium chloride presented negative deviations to ideality when mixed with tetraethylammonium chloride or tetrapropylammonium chloride, leading to a strong decrease of the melting points of these salt mixtures, in a behavior akin to that observed in the formation of deep eutectic solvents. To better rationalize this unexpected melting point depression between two structurally similar compounds devoid of dominant hydrogen bonding capability, new solid–liquid equilibria data for tetramethylammonium-based systems were measured and analyzed in this work. Molecular dynamics was used to show that the strong negative deviations from ideality presented by these systems arise from a synergetic share of the chloride ions. A transfer of chloride ions seems to occur from the bigger cation in the mixture (which possesses a more disperse charge) to the smaller cation (tetramethylammonium), resembling the formation of metal–chloride complexes in type I deep eutectic solvents. This rearrangement of the charged species leads to an energetic stabilization of both components in the mixture, inducing the negative deviations to the ideality observed. The conclusions presented herein emphasize the often-neglected contribution of charge delocalization in deep eutectic solvents formation and its applicability toward the design of new ionic liquid mixtures.

## 1. Introduction

Ionic liquids (ILs) are ionic solutions composed of non-symmetric ions with low lattice energy that are liquid below an arbitrarily accepted threshold of 373 K. ILs are often referred to as “designer solvents” due to their wide range of accessible properties, which can be freely modulated by careful selection of the anion and cation [1]. These tuneable solvents are of interest for a wide range of applications, including synthesis, catalysis [1], energy storage [2], metal extraction and separation [3], carbon capture [4], and biomass dissolution [5] amongst others. A natural extension of the design versatility of ILs is the further fine-tuning of their physicochemical properties through the use of binary mixtures of these substances, which are also referred to as eutectic ILs [6,7,8,9,10,11]. For a given temperature, eutectic solvents (ES) may be defined as liquid mixtures, usually binary, composed of substances that when pure are often solid at that temperature. A prefix *deep* is used to emphasize large negative deviations from thermodynamic ideality, resulting in a lower than predicted eutectic temperature [12]. 

Mixtures of two salts can allow for properties intermediate between those of the respective components in the case of a thermodynamically ideal mixture or properties widely different from those of the pure components in the case of non-ideal mixtures [12]. The molecular structure of IL mixtures is primarily determined by the distribution of ions driven by Coulombic interactions, with secondary contributions from hydrogen bonding and π interactions (for ions containing aromatic moieties). To date, most of the physicochemical properties of IL mixtures such as conductivity, viscosity, density, phase behavior, and thermal stabilities follow ideal or quasi-ideal mixing laws with the exceptions of mixtures with large steric effect, where the differences in ion sizes, and therefore charge densities, can interfere with the organization [6,7,8,13,14,15,16]. 

Another important exception of mixtures presenting strong deviations from ideality is in the case where a chemical reaction occurs, such as complex ions in halogenometalate ILs [7] and Type 1 deep eutectic solvents (DES) [17]. Type 1 DES comprises mixtures of quaternary ammonium salts and metal chloride salts (MClx) [17,18,19]. In these mixtures, excess chloride ions from the quaternary ammonium salt form complexes with the metal cation, leading to metal–chloride complex stochiometrics that are not present in the pure metal chloride salt. In turn, this chloride transfer behavior is responsible for the underlying properties of these mixtures [19]. Similarly, halogenometalate IL melts present complex phase diagrams dependent on the IL to the halogenometalate anion molar ratio. For example, mixtures of 1-ethyl-3-methyl imidazolium chloride ([C_2_C_1_im]Cl) with AlCl_3_ results in an unexpected variation in the Lewis acidity and melting temperature of the mixture based on the prevailing chloroaluminate species for a given composition. The melting point of [C_2_C_1_im]Cl/AlCl_3_ for *x*(AlCl_3_) = 0.5 is 280 K, whilst the melting point drops below 253 K for *x*(AlCl_3_) = 0.4 and *x*(AlCl_3_) = 0.6, respectively, before increasing after *x*(AlCl_3_) = 0.67 [20].

Recently [21], whilst studying the capability of cholinium chloride to lower the melting point of organic chloride-based salts, we discovered that tetramethylammonium chloride presented severe deviations from thermodynamic ideality when mixed with tetraethylammonium chloride and tetrapropylammonium chloride. Based on the current prevailing understanding of dominating forces in eutectic solvents, such behaviour is unexpected, since these compounds are structurally very similar and are usually not regarded as strong hydrogen bond acceptors. As a matter of fact, due to the negative deviations to ideality, these systems could even be regarded as true DES. A connection can be made between the tetramethylammonium-based eutectic ILs and type I DES by considering the replacement of the metal cation M by the tetramethylammonium cation. To the best of our knowledge, such systems would represent the first example of a fully organic-based type I DES with potential applicability, for example, as organic reaction medium where similar DES were shown to outperform conventional ILs and organic solvents [22,23,24]. In this work, we attempt to rationalize the underlying mechanism behind the formation of these tetramethylammonium chloride-based eutectic ILs through the analysis of solid–liquid equilibrium data and molecular dynamics simulations. These novel mixtures bridge the gap between conventional DES and eutectic ILs, emphasizing the often overlooked electrostatic contribution to the thermodynamic behavior of the final mixture. 

## 2. Results

**Figure 1:**
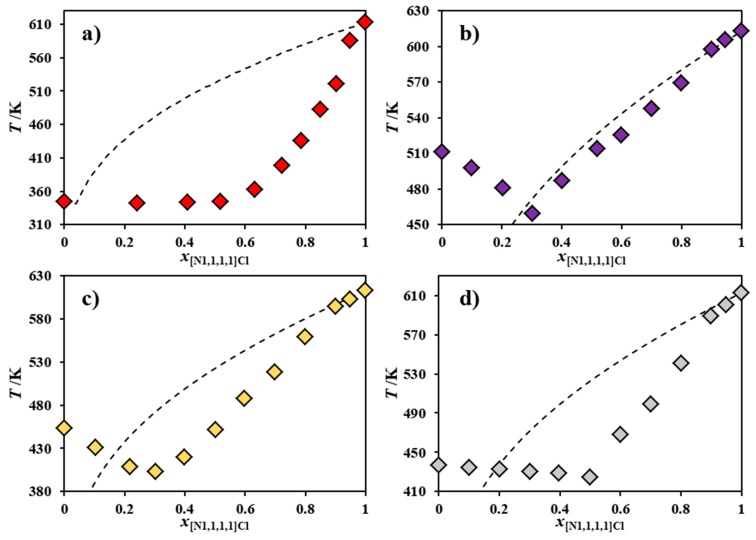
Experimental solid–liquid equilibrium phase diagram for the binary systems composed of [N_1,1,1,1_]Cl and (**a**) [N_4,4,4,4_]Cl, (**b**) [N_Bz,1,1,1_]Cl [21], (**c**) [N_Bz,2,2,2_]Cl and (**d**) [N_Bz,4,4,4_]Cl. The ideal solid–liquid equilibrium phase diagram is also included, in each case, as a dashed line.

The experimentally measured SLE phase diagrams of four [N_1,1,1,1_]Cl-based mixtures with [N_4,4,4,4_]Cl, [N_Bz,1,1,1_]Cl, [N_Bz,2,2,2_]Cl, and [N_Bz,4,4,4_]Cl are reported in Figure 1 and Table S1 of the Supporting Information, along with the corresponding ideal solubility curves, calculated (when possible) as per Equation (2) and using the properties presented in Table 2. The chemical structure of the salts studied in this work along with their relevant thermodynamic properties are presented in the Methodology section. Thermogravimetric analysis (TGA) of the pure starting components and ^1^H-NMR of the eutectic mixtures are available in Figures S1–S6 of the Supporting Information.

Even though the entire ideal SLE phase diagram could not be calculated for most of the systems reported in Figure 1, since the melting enthalpy of their second component is not available due to decomposition upon melting, it is patent that [N_1,1,1,1_]Cl presents strong negative deviations from ideality (observed as melting depressions of at least 50 K) when mixed with [N_4,4,4,4_]Cl (Figure 1a), [N_Bz,2,2,2_]Cl (Figure 1c) or [N_Bz,4,4,4_]Cl (Figure 1d). As such, these salt mixtures, in spite of their high melting points, could be classified as *deep* eutectic solvents in the sense that their experimental melting curves are much steeper than the corresponding ideal melting curves. The nature of the interactions between these compounds that lead to such non-ideal behavior is quite unexpected, taking into consideration their structural similarity.

**Figure 2:**
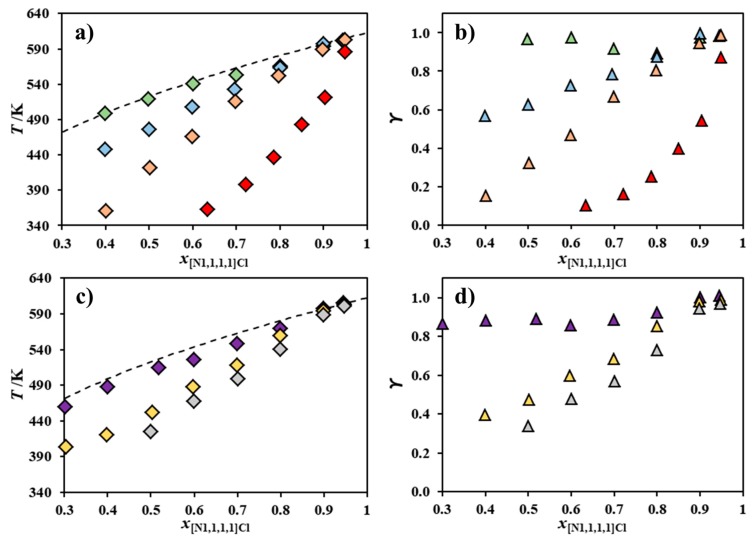
Melting curve of [N_1,1,1,1_]Cl (◇: **a**,**c**) and its activity coefficients (△: **b**,**d**) for the binary systems composed of [N_1,1,1,1_]Cl and [NH_4_]Cl [21] (◆,▲), [N_2,2,2,2_]Cl [21] (◆,▲), [N_3,3,3,3_]Cl [21] (◆,▲), [N_4,4,4,4_]Cl (◆,▲) [N_Bz,1,1,1_]Cl [21] (◆,▲), [N_Bz,2,2,2_]Cl (◆,▲), or [N_Bz,4,4,4_]Cl (◆,▲). Data for mixtures of [N_1,1,1,1_]Cl and symmetric and benzyl alkylammonium salts are presented in the upper (**a**,**b**) and lower two panels (**c**,**d**), respectively. The ideal melting curve of [N_1,1,1,1_]Cl is included as a dashed line.

Figure 2 plots the melting curves and activity coefficients of [N_1,1,1,1_]Cl in the chloride-based systems reported in Figure 1 and in the systems composed of [N_1,1,1,1_]Cl and [N_Bz,1,1,1_]Cl, [NH_4_]Cl, [N_2,2,2,2_]Cl, or [N_3,3,3,3_]Cl, which were previously published [21]. For visual simplicity, symmetric alkylammonium and benzyl alkylammonium systems are reported in separate diagrams.

Both the symmetric alkylammonium and non-symmetric benzyl alkylammonium series shown in Figure 2 reveal that the negative deviations from ideality presented by [N_1,1,1,1_]Cl appear to correlate well with the length of the alkyl chains of the second component cation; that is, as the size asymmetry between the two cations increases, [N_1,1,1,1_]Cl behaves less ideally. Interestingly, the introduction of a benzyl moiety does not appear to significantly influence the deviation from ideality observed. The systems [N_1,1,1,1_]Cl + [NH_4_]Cl and [N_1,1,1,1_]Cl + [N_Bz,1,1,1_]Cl are mostly thermodynamically ideal, whilst [N_1,1,1,1_]Cl presents severe negative deviations from ideality in the systems [N_1,1,1,1_]Cl + [N_4,4,4,4_]Cl and [N_1,1,1,1_]Cl + [N_Bz,4,4,4_]Cl. Negative deviations from ideality are present when a given component possesses stronger interactions with the component it is mixed with, rather than with itself in the pure state. The strong attractive forces required to justify the observed large negative deviations are upon first reflection counter-intuitive, considering that both salts share a common anion, whilst electrostatic repulsion should hinder any favorable interaction between the cations of the components of these mixtures. Thus, it is here hypothesized that negative deviations from ideality can only arise from a synergetic share of the total available chlorides in these mixtures. Since the [N_1,1,1,1_]^+^ cation possesses a more electropositive surface than its counterparts ([N_4,4,4,4_]^+^, for instance), chloride anions should prefer to interact with it, leading to an energetic stabilization of both components and the mixture itself. This is analogous to type I deep eutectic solvents, where chloride ions are transferred to the metallic cation, originating metal–chloride complexes [17,18,19,25].

The chloride transfer hypothesis introduced above also explains why the length of the alkyl chains in the cation of the second component correlates with the magnitude of the negative deviations from ideality presented by [N_1,1,1,1_]Cl. As the second component cation alkyl chain length increases, its central charged nitrogen becomes less accessible, leading to a weaker interaction with chloride. As such, the chloride becomes easier to transfer to the less bulky cation—in this case, [N_1,1,1,1_]^+^, which possesses available space to interact with it. Besides the decrease of the anion–cation interaction, the increase of cation alkyl chain length may also lead to less unfavorable cation–cation interactions through the minimization of Coulombic repulsion due to stronger London dispersive forces between their alkyl groups. This effect would positively add to the decrease in strength of the anion–cation interaction, further prompting the chloride transfer to [N_1,1,1,1_]Cl.

**Figure 3:**
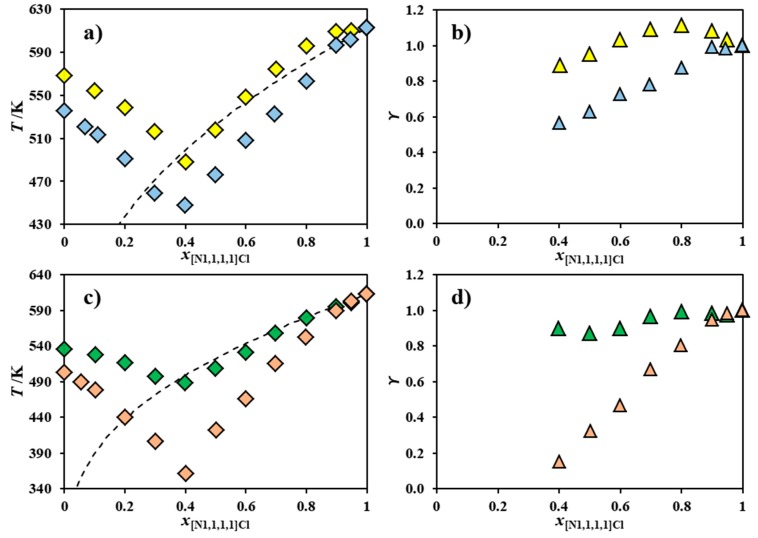
Experimental solid–liquid equilibrium (SLE) phase diagrams (◇: **a**,**c**) of the systems composed of (**a**) [N_1,1,1,1_]Cl and [N_2,2,2,2_]Cl [21] (◆) or [N_2,2,2,2_]Br (◆) and composed of (**c**) [N_1,1,1,1_]Cl and [N_3,3,3,3_]Cl [21] (◆) or [N_3,3,3,3_]Br (◆). The ideal melting curve of [N_1,1,1,1_]Cl (**- - -**) and its activity coefficients (△: **b**,**d**) are also included.

To test the chloride transfer hypothesis, Figure 3 shows the SLE phase diagrams of the binary systems [N_1,1,1,1_]Cl and [N_2,2,2,2_]Cl [21] or [N_2,2,2,2_]Br and of the systems [N_1,1,1,1_Cl] and [N_3,3,3,3_]Cl [21] or [N_3,3,3,3_]Br, along with the corresponding activity coefficients. Since the bromide anion is larger and less electronegative than chloride, its transfer from the second component to the [N_1,1,1,1_]Cl should be more difficult and less energetically favorable. Figure 3 reveals a significant difference in the behavior of [N_1,1,1,1_]Cl when mixed with either a chloride-based salt or a bromide-based salt. Figure 3a shows that [N_1,1,1,1_]Cl presents negative deviations from ideality when mixed with [N_2,2,2,2_]Cl, but a near-ideal behavior when mixed with [N_2,2,2,2_]Br. The systems shown in Figure 3c show a similar behavior: [N_1,1,1,1_]Cl presents negative deviations from ideality when mixed with [N_3,3,3,3_]Cl, but behaves ideally when mixed with [N_3,3,3,3_]Br. All of this supports the chloride transfer hypothesis, especially considering the secondary contribution to non-ideality of the hydrogen bonding between [N_1,1,1,1_]^+^ and Cl^−^ compared to [N_1,1,1,1_]^+^ and Br^−^. Previous density functional theory calculations of the choline chloride ([N_1,1,1,2OH_]Cl) ion pair revealed the relatively strong hydrogen bond interactions between three methyl moieties around the cholinium nitrogen center and the chloride anion [26]. Similar interactions are envisaged between [N_1,1,1,1_]^+^ and Cl^−^ and decrease upon the substitution of this anion by bromide due to the decrease of the latter’s electronegativity.

**Figure 4:**
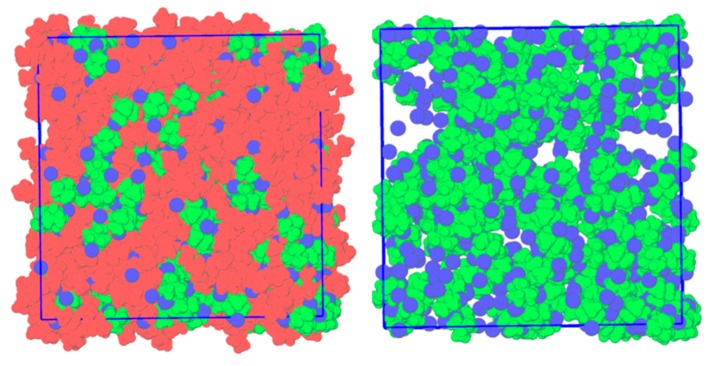
**Right**: Final simulation snapshot of the liquid-phase equimolar mixture of [N_1,1,1,1_]Cl and [N_4,4,4,4_]Cl at 353 K. **Left**: Identical system with [N_4,4,4,4_]^+^ removed to better identify the presence of [N_1,1,1,1_]^+^ − Cl^−^ clusters. Color scheme: green for [N_1,1,1,1_]^+^, red for [N_4,4,4,4_]^+^, and blue for Cl^−^.

To further understand the relationship between chloride transfer and the negative deviations to ideality observed, MD simulations of equimolar liquid-phase mixtures of [N_1,1,1,1_]Cl with [N*_x,x,x,x_*]Cl (*x* = 2–4) were computed and compared to the pure hypothetical liquid phases of [N*_x,x,x,x_*]Cl. The simulation temperatures are listed for each system in Table 1, and the final simulation snapshot of the liquid-phase equimolar mixture of [N_1,1,1,1_]Cl + [N_2,2,2,2_]Cl, [N_1,1,1,1_]Cl + [N_3,3,3,3_]Cl and [N_1,1,1,1_]Cl + [N_4,4,4,4_]Cl is presented in Figure 4 and Figure S7 of the Supporting Information. To better appreciate the molecular-scale segregation occurring in mixtures of structurally similar compounds with significant differences in molar volumes, a domain analysis was performed based on the Voronoi tessellation method. The system was divided into three subsets ([N_1,1,1,1_]^+^, [N*_x,x,x,x_*]^+^ for *x* = 2–4 and {[N_1,1,1,1_]^+^ + Cl^−^}), and the average number of domains of each subset during the simulation was calculated with the results presented in Table 1. A value of 1 implies the molecules in a given subset form a continuous aggregate, whilst a larger value is indicative of a dispersed subset. In the [N_1,1,1,1_]Cl + [N*_x,x,x,x_*]Cl (*x* = 2–4), the larger tetraalkylammonium cations overcome their electrostatic repulsion to form a single domain. The presence of longer alkyl moieties shielding the localized charge on the nitrogen center results in more prominent dispersive interactions and the formation of a continuous ‘apolar domain’ compared to [N_1,1,1,1_]^+^ + Cl^−^ rich regions. In contrast, {[N_1,1,1,1_]^+^ + Cl^−^} form discreet charge-dense aggregates, with the aggregation number linearly increasing with the ionic radius of the counter-cation from [N_2,2,2,2_]^+^ to [N_4,4,4,4_]^+^ (Table 1). This is particularly evident in the snapshot of the equilibrated [N_1,1,1,1_]Cl + [N_4,4,4,4_]Cl system (Figure 4), in which negatively charged non-stochiometric {[N_1,1,1,1_]^+^ + Cl^−^} aggregates can be observed amidst a low-polarity and chloride-deficient [N_4,4,4,4_]^+^ pseudo-phase. It is important to stress that this segregation does not represent a phase separation, as the mixture is fully miscible. This extra structuring in the mixtures increases the rigidity of the system. The diffusion constant of the different components in the liquid mixtures and pure ion pairs were calculated using the mean square displacement (MSD) method and reported in Table S2 of the Supporting Information. In all three systems, the diffusion coefficients of [N*_x,x,x,x_*]^+^ (*x* = 2–4) and Cl^−^ were reduced in the eutectic mixtures compared to the pure liquid systems at a given temperature.

**Figure 5:**
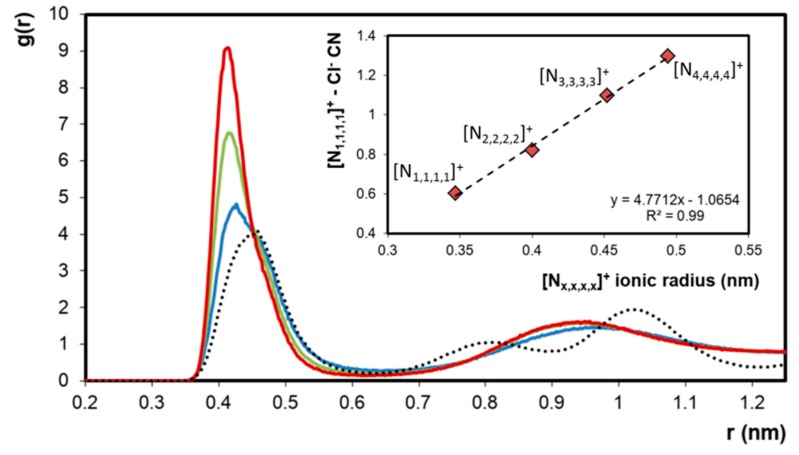
Radial distribution functions (RDFs) of chloride around the [N_1,1,1,1_]^+^ cation in the corresponding chloride–salt hypothetical pure liquid phase (dashed line) and in the equimolar liquid phase mixtures of [N_1,1,1,1_]Cl + [N_2,2,2,2_]Cl (──), [N_1,1,1,1_]Cl + [N_3,3,3,3_]Cl (──) and [N_1,1,1,1_]Cl + [N_4,4,4,4_]Cl (──). In each case, the x-axis represents the distance of the chloride ion from the central nitrogen of the cation. Inset: Chloride coordination number (CN) in the presented systems at r = 0.414 nm as a function of the ionic radius of the largest component in the system [27].

The apparent segregation of [N_1,1,1,1_]Cl + [N*_x,x,x,x_*]Cl (*x* = 2–4) liquid mixtures into chloride-rich and chloride-poor regions was further interpreted using RDFs and compared to that obtained in liquid single ion-pair systems. Such comparisons are only theoretically possible as the smaller tetralkylammonium chloride salts degrade upon melting. The RDFs of chloride around the [N_1,1,1,1_]^+^ cation in the three eutectic mixtures studied as well as in the liquid [N_1,1,1,1_]Cl system (*T* = 633 K, dotted line) are presented in Figure 5. The computed RDFs clearly indicate the enhanced presence of chloride ions around [N_1,1,1,1_]^+^ when mixed with [N*_x,x,x,x_*]Cl (*x* = 2–4) compared to its pure liquid state. Furthermore, the extent of chloride transfer to [N_1,1,1,1_]^+^ is directly correlated to the size of the of the counter-cation. The coordination number (CN) of chloride around [N_1,1,1,1_]^+^ at r = 0.414 nm, corresponding to the distance to the first RDF peak maximum, is plotted as a function of the ionic radius of the larger component in the system resulting in a linear correlation (Figure 5). These results show that when [N_1,1,1,1_]Cl and [N*_x,x,x,x_*]Cl (*x* = 2–4) are mixed, a chloride transfer occurs from the larger [N*_x,x,x,x_*]^+^ cation to the [N_1,1,1,1_]^+^ cation, the extent of which is determined by the difference in ionic radius between the two cations. The observed chloride enrichment around [N_1,1,1,1_]^+^ engenders a corresponding chloride depletion around [N*_x,x,x,x_*]^+^ compared to the pure compound, as evidenced in Figures S8–S10 of the Supporting Information. 

**Figure 6:**
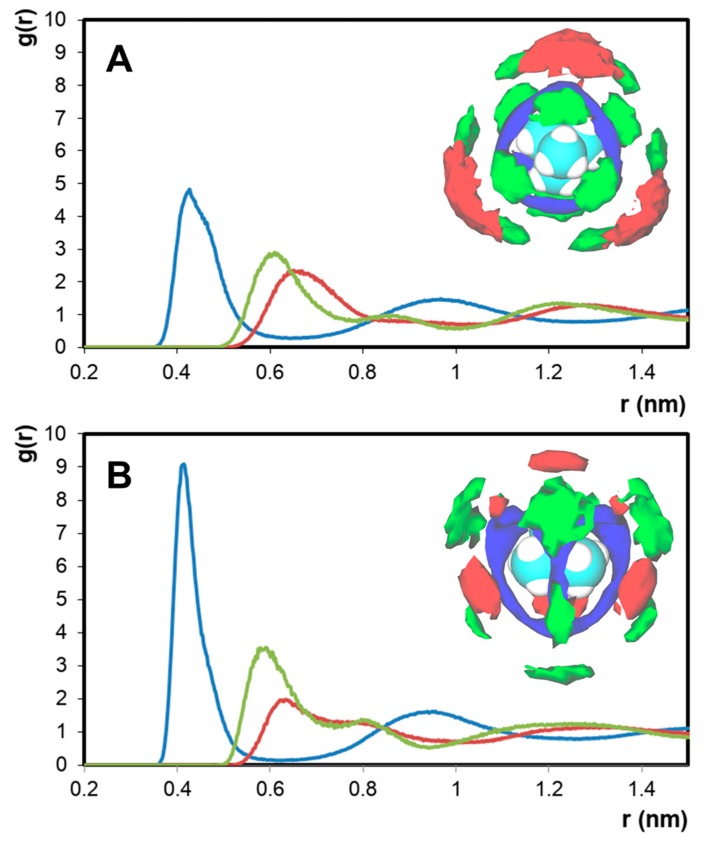
RDFs of chloride, [N_1,1,1,1_]^+^, and [N_x,x,x,x_]^+^ around the [N_1,1,1,1_]^+^ cation in the system. (**A**) [N_1,1,1,1_]Cl + [N_2,2,2,2_]Cl and (**B**) [N_1,1,1,1_]Cl + [N_4,4,4,4_]Cl. In each case, the x-axis represents the distance from the central nitrogen of [N_1,1,1,1_]^+^. Inset: Corresponding SDF analysis of the system with reference to the [N_1,1,1,1_]^+^ cation. Color scheme: green for [N_1,1,1,1_]^+^, red for [N_x,x,x,x_]^+^, and blue for Cl^−^.

The impact of the {[N_1,1,1,1_]^+^ − Cl^−^} structuring on the nanosegregation of the studied system was further probed by calculating the RDFs and SDFs of the different system components in the [N_1,1,1,1_]Cl + [N_2,2,2,2_]Cl and [N_1,1,1,1_]Cl + [N_4,4,4,4_]Cl mixtures with respect to [N_1,1,1,1_]^+^ (Figure 6). RDF analysis in both systems suggests a similar structural ordering with regard to the reference [N_1,1,1,1_]^+^ cation. The primary solvation shell of [N_1,1,1,1_]^+^ is composed of chloride anions acting as an electrostatic bridge with other [N_1,1,1,1_]^+^ molecules. The longer range intercationic interaction [N_1,1,1,1_]^+^ − [N_4,4,4,4_]^+^ is present at r = 0.63 nm, after the [N_1,1,1,1_]^+^ − [N_1,1,1,1_]^+^ peak at r = 0.58 nm, suggesting the dominance of ([N_1,1,1,1_]Cl_(1+z)_)^−z^ complexes on the liquid structuring. The decreasing intensity of the [N_1,1,1,1_]^+^ – [N*_x,x,x,x_*]^+^ peak with increasing [N*_x,x,x,x_*]^+^ ionic radius is not matched by an increase in [N_1,1,1,1_]^+^ – [N_1,1,1,1_]^+^ interaction but rather by [N_1,1,1,1_]^+^ − Cl^−^, further confirming chloride delocalization as the governing factor in the observed non-ideality. SDF analysis shows the difference in [N_1,1,1,1_]^+^ – [N*_x,x,x,x_*]^+^ interactions with decreasing [N*_x,x,x,x_*]^+^ charge density. In the secondary solvation shell of [N_1,1,1,1_]^+^, [N_2,2,2,2_]^+^ competes with other [N_1,1,1,1_]^+^ cations for Cl^−^ interaction, as suggested by the stacking of the [N_1,1,1,1_]^+^ (green) and [N_2,2,2,2_]^+^ (red) isosurfaces with that of Cl^−^ (blue). In contrast, the bulky [N_4,4,4,4_]^+^ cation preferentially interacts with [N_1,1,1,1_]^+^ through its methyl moieties, allowing for the formation of an apolar [N_4,4,4,4_]^+^ pseudo-phase with well-dispersed ([N_1,1,1,1_]Cl_(1+z)_)^−z^ aggregates and resulting in a large deviation from ideality.

This rearrangement of chloride ions in [N_1,1,1,1_]Cl/[N_x,x,x,x_]Cl mixtures leads to an energetic stabilization of both [N_1,1,1,1_]Cl and [N*_x,x,x,x_*]Cl, originating the remarkable negative deviations from ideality presented by [N_1,1,1,1_]Cl in these systems. Such a simplified explanation is possible due to the absence of dominant hydrogen-bonding functionalities. The dominance of [N_1,1,1,1_]^+^ − Cl^−^ interactions and the formation of negatively charged ([N_1,1,1,1_]Cl_(1+z)_)^−z^ oligomers bears, as mentioned, resemblance to metal–chloride complexes in type I deep eutectic solvents [17,18,19,25], as well as to the ionic structuring observed in “type IV” metal-based eutectics [28], halometallates ionic liquids [29], and mixtures of chloride molten salts with cations of differing charge densities [30,31]. For example, the occurrence of an anionic chloro–nickel complex NiCl_4_^2−^ in molten salt mixtures with alkali chloride becomes increasingly evident as the size of the alkali cation is increased from Li to Cs [30]. Such ionic structuring greatly differs from the reported hydrogen bond-dominated liquid structure of conventional cholinium chloride-based eutectic solvents [32]. The conclusions presented herein emphasize the often-neglected contribution of charge delocalization in DES formation and its applicability toward the formation of new eutectic ionic liquids.

## 3. Materials and Methods 

### 3.1. Experimental Details

#### 3.1.1. Chemicals

**Figure 7:**
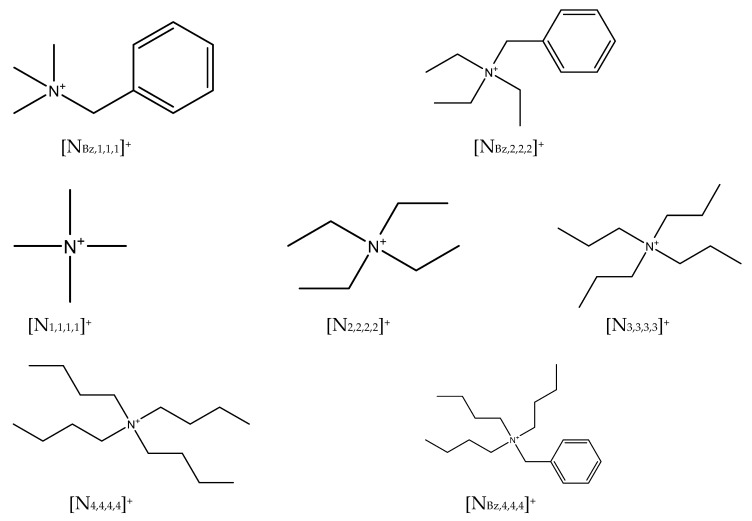
Chemical structure of the cation of each halide-based salt experimentally studied in this work.

In this work, the salts tetramethylammonium chloride ([N_1,1,1,1_]Cl), tetrabutylammonium chloride ([N_4,4,4,4_]Cl), benzyltrimethylammonium chloride ([N_Bz,1,1,1_]Cl), benzyltriethylammonium chloride ([N_Bz,2,2,2_]Cl), benzyltributylammonium chloride ([N_Bz,4,4,4_]Cl), tetraethylammonium bromide ([N_2,2,2,2_]Br), and tetrapropylammonium bromide ([N_3,3,3,3_]Br) were experimentally used. Their CAS number, supplier, purity, and melting properties are reported in Table 2, while the chemical structure of their cations is depicted in Figure 7. Since the salts listed above are hygroscopic, they were carefully dried under vacuum (0.1 Pa) and constant stirring at room temperature (298 K) for at least 72 h prior to use. The water content of the salts after drying was verified using a Metrohm 831 Karl Fischer coulometer, with the analyte Hydranal Coulomat AG from Riedel-de-Haen, and is presented in Table 2. 

#### 3.1.2. Experimental Protocol and Characterization

For each [N_1,1,1,1_]Cl-based binary system, several mixtures were prepared covering its entire composition range. They were prepared inside a dry-argon glove box to prevent contamination by air humidity. The proper amount of each pure component was weighted using an analytical balance (model ALS 220-4N from Kern) with an accuracy of ±0.002 g. After preparation, each sample was molten and recrystallized in order to maximize the contact between the two components. Then, still inside the glove box, the samples were crushed in a mortar, and part of the resulting powder was used to fill glass capillaries. The melting point of the sample inside each glass capillary was measured using the melting point device model M-565 from Buchi, with a temperature resolution of 0.1 K and a temperature gradient of 0.1 K·min^−1^. This procedure was repeated at least three times, and all the measurements have an estimated reproducibility better than 1.9 K (see Table S1 of the Supporting Information).

The melting temperature and enthalpy of [N_4,4,4,4_]Cl was measured using differential scanning calorimetry (DSC) in a Hitachi DSC7000X model working at atmospheric pressure. Samples of approximately 2–5 mg tightly sealed in aluminium pans were prepared inside the dry-argon glove box and submitted to three repeated cooling–heating cycles at 5 K·min^−1^ (cooling) and 2 K·min^−1^ (heating). The thermal transition temperature was taken as the peak temperature. The equipment was calibrated with several standards (heptane, octane, decane, 4-nitrotoluene, naphthalene, benzoic acid, diphenylacetic acid, indium, tin, caffeine, lead, zinc, potassium nitrate, water, and anthracene) with weight fraction purities higher than 99%. The melting temperature of [N_4,4,4,4_]Cl was previously reported with a value of 348 K [34]. Due to their degradation upon melting, it was not possible to measure the melting properties of the salts [N_Bz,1,1,1_]Cl, [N_Bz,2,2,2_]Cl, [N_Bz,4,4,4_]Cl, [N_2,2,2,2_]Br, or [N_3,3,3,3_]Br using this methodology. The degradation temperatures of the ammonium salts were obtained using a Setsys Evolution 1750 (SETARAM) instrument under nitrogen atmosphere with a heating rate of 10 K·min^−1^ (precision: temperature ±0.01 K; mass ±0.01 mg). TGA thermograms are presented in Figure S1 of the Supporting Information, and the onset and peak degradation temperatures are listed in Table 2. These are in good agreement with the values reported in the literature: [N_1,1,1,1_]Cl, 633 K [35]; [N_4,4,4,4_]Cl, 473 K (obtained from supplier Molbase); [N_Bz,1,1,1_]Cl, 514-516 K (obtained from supplier CDH Fine Chemicals); [N_Bz,2,2,2_]Cl, 463–465 K (obtained from the European Chemicals Agency); [N_Bz,4,4,4_]Cl, 428 K (obtained from supplier ChemLabs); [N_2,2,2,2_]Br, 558 K [36]; [N_3,3,3,3_]Br, 543 K [36].

Due to the proximity of the melting and degradation temperatures for most of the salts listed in Table 2, ^1^H-NMR analysis of the different mixtures at their eutectic composition were performed after heating and recrystallization to verify the extent of degradation. All samples were rapidly heated under agitation until a homogenous solution was observed, cooled immediately thereafter, and analyzed 24 h after their formation. The ^1^H-NMR spectra of the single salts were also determined for comparison. All ^1^H-NMR spectra were recorded at room temperature using a Bruker Avance 300 operating at 75 MHz with deuterated water as solvent, and are presented in Figures S2–S6 of the Supporting Information. No differences in the spectra or new NMR signals were observed for mixtures composed of [N_1,1,1,1_]Cl and [N_2,2,2,2_]Cl, [N_3,3,3,3_]Cl, [N_4,4,4,4_]Cl, [N_2,2,2,2_]Br, [N_3,3,3,3_]Br, or [N_Bz,1,1,1_]Cl when compared with the pure components, indicating that no decomposition products are formed. For mixtures containing [N_Bz,2,2,2_]Cl and [N_Bz,4,4,4_]Cl, a yellowing of the solution was observed, and ^1^H-NMR analysis of the [N_1,1,1,1_]Cl + [N_Bz,2,2,2_]Cl mixture (Figure S6 of the Supplementary Information) indicated approximately 20 mol.% decomposition. The [N_1,1,1,1_]Cl + [N_Bz,4,4,4_]Cl mixture could not be analyzed by NMR, as its decomposition products were only partially soluble in deuterated water, DMSO, and chloroform, but is expected to present a similar decomposition extent as the [N_1,1,1,1_]Cl + [N_Bz,2,2,2_]Cl mixture. **Therefore, we advise caution on the part of the reader when considering the reported data on these two mixtures**.

### 3.2. Computational Details

#### 3.2.1. Thermodynamic Framework

When the individual components of an eutectic-type liquid mixture solidify into a pure form (i.e., complete solid-phase immiscibility), their solid–liquid equilibrium (SLE) curves are described by the equation [37,38]:

(1) $$\ln\left(x_{i}\cdot \gamma_{i}\right)=\frac{\Delta_{m}h_{i}}{R}\cdot \left(\frac{1}{T_{m,i}}-\frac{1}{T}\right)+\frac{\Delta_{m}Cp_{i}}{R}\cdot \left(\frac{T_{m,i}}{T}-ln\frac{T_{m,i}}{T}-1\right)$$

where $x_{i}$ is the mole fraction of the general component i, $\gamma_{i}$ is its activity coefficient, $\Delta_{m}h_{i}$ is its melting enthalpy, $T_{m,i}$ is its absolute melting temperature, and $\Delta_{m}Cp_{i}$ is its melting heat capacity change, while T is the absolute temperature of the system, and R is the ideal gas constant. Due to the scarce data in the literature regarding the physical constant $\Delta_{m}Cp_{i}$, which is mostly related to the difficulty of its measurement, and its negligible impact in the final SLE curve, Equation (1) may be simplified to [38,39]:

(2) $$\ln\left(x_{i}\cdot \gamma_{i}\right)=\frac{\Delta_{m}h_{i}}{R}\cdot \left(\frac{1}{T_{m,i}}-\frac{1}{T}\right)$$

Equation (2) is useful, since it allows the prediction of the ideal SLE phase diagram of a mixture by setting the activity coefficients of all components equal to unity. This equation can also be used to calculate the activity coefficients of the components in a mixture from their experimental SLE curves.

#### 3.2.2. Molecular Dynamics Simulations

Molecular dynamic simulations were carried out using Gromacs 5.1 package [40] within the NpT ensemble by adopting the leapfrog algorithm to integrate the equations of motion at a fixed temperature and pressure (1 bar) [41]. The force field parameters of symmetrical quaternary ammonium chloride salts were taken from the modified OPLS-AA all-atom force field developed by Canongia Lopes et al. [42,43]. Hydrogen bonds were constrained by the LINCS algorithm [44] whilst Lennard-Jones (LJ) and Coulombic interactions were computed up to a cut-off radius of 1.2 nm. The force-switch van der Waals potential modifier was employed for LJ, where the energy decays smoothly to zero between 0.9–1.2 nm, while long-range Coulombic interactions were evaluated by particle mesh Ewald (PME) [45]. All simulations contained 512 ion pairs initially randomly distributed in a cubic box, and production runs were carried for 60 ns with a time step of 2 fs following an energy minimization step using the steepest descent algorithm and two short equilibrium runs in the NVT and NpT ensembles, respectively. The temperature and pressure (fixed at 1 bar) were controlled through the Nose–Hoover thermostat [46] and the Parrinello–Rahman barostat [47], respectively. The optimized geometry of the starting components were obtained by density functional theory (DFT) calculations using Gaussian 09 [48] employing Becke’s three-parameter exchange [49] in combination with the Lee, Yang, and Parr correlation functional (B3LYP) [50]. All elements were computed at the B3LYP/6-311+G(d,p) level of theory. Atomistic charges were determined using the CHELPG methodology [51] and scaled by 0.9, which is in line with previously reported studies on organic eutectic mixtures [52]. Simulations of the equimolar [N_1,1,1,1_]Cl + [N_4,4,4,4_]Cl system for various charge scaling from 0.8 to 1.0 indicates that charge scaling did not affect the extent of chloride transfer, which is the parameter of interest in this study, but did have an influence on the physical properties of the mixture. MD simulation outputs were visualized using the VMD software package [53]. Radial distribution functions (RDFs) and diffusion coefficients were calculated using Gromacs inbuilt analysis tools. Spatial distribution functions (SDF) and domain analyses based on radical Voronoi tessellation were obtained using the TRAVIS software package [54,55].

## 4. Conclusions

In this work, the unexpected behavior of tetramethylammonium-based systems with other quaternary ammonium salts was investigated, and new solid–liquid equilibria data were measured and reported. These systems present strong negative deviations from ideality despite their structural similarities and lack of strong hydrogen bonding capabilities. By replacing the chloride ion with a bromide ion, the negative deviations from ideality were no longer present and the components in these mixtures behaved ideally. Thus, a chloride transfer hypothesis was put forward and supported by molecular dynamics simulations of these systems. Molecular dynamic simulations indicate that the experimentally observed abnormal negative deviations from ideality in quaternary ammonium-based mixtures results from a synergetic share of the chloride ions present. The chloride ions are preferentially found in the vicinity of the smaller cation present in the mixture (tetramethylammonium cation), leading to an energetic stabilization of both components. This effect was shown to be similar to what has been observed in metal-based deep eutectic solvents of types I and IV, as well as other metal-based mixtures. This work reports the feasibility of using tetramethylammonium chloride as a replacement for metal chloride salts to form new type I deep eutectic solvents, or, in a more general view, the feasibility of preparing quaternary ammonium-based eutectic ionic liquids. Moreover, the charge delocalization present in these mixtures may be altered through a judicious choice of their composition, leading to a fine-tuning of the polarity of the resulting solvent.

## Figures and Tables

**Table 1:** Domain analysis of the liquid-phase equimolar mixtures of [N_1,1,1,1_]Cl with [N_x,x,x,x_]Cl (x = 2–4).

System	*T* (K)	[N_x,x,x,x_]^+^ Ionic Radii (nm) [27]	Domain Analysis		
			[N_1,1,1,1_]^+^	[N_1,1,1,1_]^+^ + Cl^−^	[N*_x_*_,*x*,*x*,*x*_]^+^
[N_1,1,1,1_]Cl + [N_2,2,2,2_]Cl	533.15	0.400	1.7	15.3	1.0
[N_1,1,1,1_]Cl + [N_3,3,3,3_]Cl	423.15	0.452	6.0	30.2	1.0
[N_1,1,1,1_]Cl + [N_4,4,4,4_]Cl	353.15	0.494	5.3	48.5	1.0

**Table 2:** CAS number, supplier, purity, melting temperature (*T_m_*), degradation temperature (*T_deg_*), water content, and melting enthalpy (Δ*_m_H*) for the ionic substances experimentally studied in this work.

Substance	CAS Number	Supplier	Purity/wt.%	*T_m_*/K	*T_deg_* (onset/peak)/K	[H_2_O]/wt.%	Δ*_m_H*/kJ·mol^−1^
[N_1,1,1,1_]Cl	75-57-0	Sigma-Aldrich	97	612.9 [22]	602.3/627.5	0.30	20.49 ^c^
[N_4,4,4,4_]Cl	1112-67-0	Sigma-Aldrich	97	344.0 *^a^*	453.7/484.1	0.58	14.69 *^a^*
[N_Bz,1,1,1_]Cl	56-93-9	Acros Organics	98	511.0 *^b^*	512.1/519.1	1.26	^_____^
[N_Bz,2,2,2_]Cl	56-37-1	Acros Organics	98	453.6 *^b^*	464.4/475.1	0.68	^_____^
[N_Bz,4,4,4_]Cl	23616-79-7	Acros Organics	98	437.0 *^b^*	441.6/452.2	0.32	^_____^
[N_2,2,2,2_]Br	71-91-0	Alfa Aesar	98	568.3 *^b^*	532.7/571.3	0.21	^_____^
[N_3,3,3,3_]Br	1941-30-6	Sigma-Aldrich	98	535.0 *^b^*	547.3/557.2	0.34	^_____^

*^a^* Measured in this work using differential scanning calorimetry; *^b^* Measured in this work using a glass capillary visual method; *^c^* Reference [33].

## Supplementary Materials

The following are available online at https://www.mdpi.com/1420-3049/24/20/3687/s1, Figure S1: TGA thermograms of the quaternary ammonium salts used in this work; Figures S2–S6: ^1^H-NMR spectra of the pure component and the eutectic mixtures at the eutectic composition (following melting and recrystallization) in deuterated water as solvent; Figure S7: Final simulation snapshot of the liquid-phase equimolar mixture of (A) [N_1,1,1,1_]Cl − [N_2,2,2,2_]Cl at 533 K, [N_1,1,1,1_]Cl − [N_3,3,3,3_]Cl at 423 K, and [N_1,1,1,1_]Cl − [N_4,4,4,4_]Cl at 353 K; Figures S8–S10: RDFs of chloride around the [N_x,x,x,x_]^+^ cation in the corresponding chloride–salt hypothetical pure liquid phase and in the equimolar liquid phase mixtures of [N_1,1,1,1_]Cl + [N_x,x,x,x_]Cl; Table S1: Experimental (x_1_,T) data and activity coefficients of the solid–liquid equilibria for eutectic mixtures composed of quaternary ammonium-based salts, at atmospheric pressure; Table S2: Diffusion coefficient (D) of the liquid phase components in the pure system and eutectic mixtures, and the ratio between the two.
