# Supplementary material for: The Role of Charge Transfer in the Formation of Type I Deep Eutectic Solvent-Analogous Ionic Liquid Mixtures
Source: Molecules. 2019 Oct 14;24(20):3687. doi: 10.3390/molecules24203687 (PMC6832115; doi:10.3390/molecules24203687)
Supplement: Supplementary file 1 [file molecules-24-03687-s001.pdf]

The Role of Charge Transfer in the Formation of Type I Deep Eutectic Solvent-Analogous Ionic Liquid Mixtures

Dinis O. Abranches[1], Nicolas Schaeffer[1], Liliana P. Silva[1], Mónia A. R. Martins[1,2,3], Simão P. Pinho[2,3] and João A. P. Coutinho[1]

[1] CICECO – Aveiro Institute of Materials, Department of Chemistry, University of Aveiro, 3810-193 Aveiro, Portugal

[2] Associate Laboratory LSRE-LCM, Instituto Politécnico de Bragança, Campus de Santa Apolónia, 5300-253 Bragança, Portugal

[3] Centro de Investigação de Montanha (CIMO), Instituto Politécnico de Bragança, Campus de Santa Apolónia, 5300-253 Bragança, Portugal

Corresponding author e-mail address: jcoutinho@ua.pt (João A. P. Coutinho).

Number of Pages: 12

Number of Figures: 10

Number of Tables: 2

Tables

Table S1. Experimental (x_1,T) data and activity coefficients of the solid-liquid equilibria for eutectic mixtures composed of quaternary ammonium-based salts, at atmospheric pressure.[a]

x_1	T /K	γ_1	x_1	T /K	γ_2
		$[N_{1,1,1,1}]Cl$ (1) + $[N_{4,4,4,4}]Cl$ (2)			
	$[N_{1,1,1,1}]Cl$ phase			$[N_{4,4,4,4}]Cl$ phase	
0.950	585.5	0.872	0.520	344.8	2.11
0.904	520.7	0.543	0.408	343.7	1.68
0.850	482.0	0.395	0.242	342.4	1.29
0.787	436.0	0.249			
0.723	398.0	0.158			
0.634	362.8	0.099			
		$[N_{1,1,1,1}]Cl$ (1) + $[N_{Bz,2,2,2}]Cl$ (2)			
	$[N_{1,1,1,1}]Cl$ phase			$[N_{Bz,2,2,2}]Cl$ pase	
0.948	603.1	0.987	0.304	402.9	——
0.900	594.2	0.979	0.218	408.7	——
0.800	559.7	0.852	0.105	431.0	——
0.700	517.9	0.684			
0.598	487.6	0.595			
0.503	451.5	0.472			
0.399	419.7	0.394			
		$[N_{1,1,1,1}]Cl$ (1) + $[N_{Bz,4,4,4}]Cl$ (2)			
	$[N_{1,1,1,1}]Cl$ phase			$[N_{Bz,4,4,4}]Cl$ phase	
0.948	600.3	0.969	0.396	428.3	——
0.900	588.7	0.942	0.303	430.6	——
0.800	540.6	0.730	0.201	432.7	——
0.701	498.6	0.567	0.102	434.5	——
0.601	467.7	0.478			
0.500	424.7	0.336			
		$[N_{1,1,1,1}]Cl$ (1) + $[N_{2,2,2,2}]Br$ (2)			
	$[N_{1,1,1,1}]Cl$ phase			$[N_{2,2,2,2}]Br$ phase	
0.949	609.7	1.03	0.300	515.9	0.923
0.899	608.8	1.08	0.201	538.5	0.986
0.800	595.7	1.11	0.101	554.2	0.997
0.699	574.4	1.09			
0.600	547.9	1.03			
0.501	517.8	0.954			
0.403	488.0	0.888			
		$[N_{1,1,1,1}]Cl$ (1) + $[N_{3,3,3,3}]Br$ (2)			

	[$N_{1,1,1,1}$]Cl phase			[$N_{3,3,3,3}$]Br phase	
0.949	600.9	0.973	0.301	496.9	
0.900	595.0	0.985	0.201	515.9	
0.800	579.6	0.991	0.103	527.6	
0.698	557.9	0.964			
0.599	531.1	0.898			
0.501	508.2	0.872			
0.399	488.5	0.899			

[a]Standard uncertainties, u, are $u(T)$ = 1.90 K, $u_r(x)$ = 0.002.

Table S2. Diffusion coefficient (D) of the liquid phase components in the pure system and eutectic mixtures and the ratio between the two.

**System**	***T* /K**	***D* × 10^{-9} (cm^2/s)**			***D*$_{eutectic}$/*D*$_{pure}$**	
		[$N_{1,1,1,1}$]$^+$	Cl^-	[$N_{x,x,x,x}$]$^+$	Cl^-	[$N_{x,x,x,x}$]$^+$
[$N_{2,2,2,2}$]Cl	533.15	-	7250 ± 540	4390 ± 121	0.52	0.52
[$N_{1,1,1,1}$]Cl + [$N_{2,2,2,2}$]Cl	533.15	2530 ± 226	3770 ± 253	2270 ± 320		
[$N_{3,3,3,3}$]Cl	423.15	-	105 ± 3.9	66.2 ± 0.9	0.74	0.69
[$N_{1,1,1,1}$]Cl + [$N_{3,3,3,3}$]Cl	423.15	51.1 ± 3.4	78.1 ± 2.6	45.5 ± 0.4		
[$N_{4,4,4,4}$]Cl	353.15	-	13.7 ± 0.4	7.10 ± 0.32	0.35	0.38
[$N_{1,1,1,1}$]Cl + [$N_{4,4,4,4}$]Cl	353.15	2.82 ± 0.28	4.76 ± 0.08	2.69 ± 0.15		

Figures

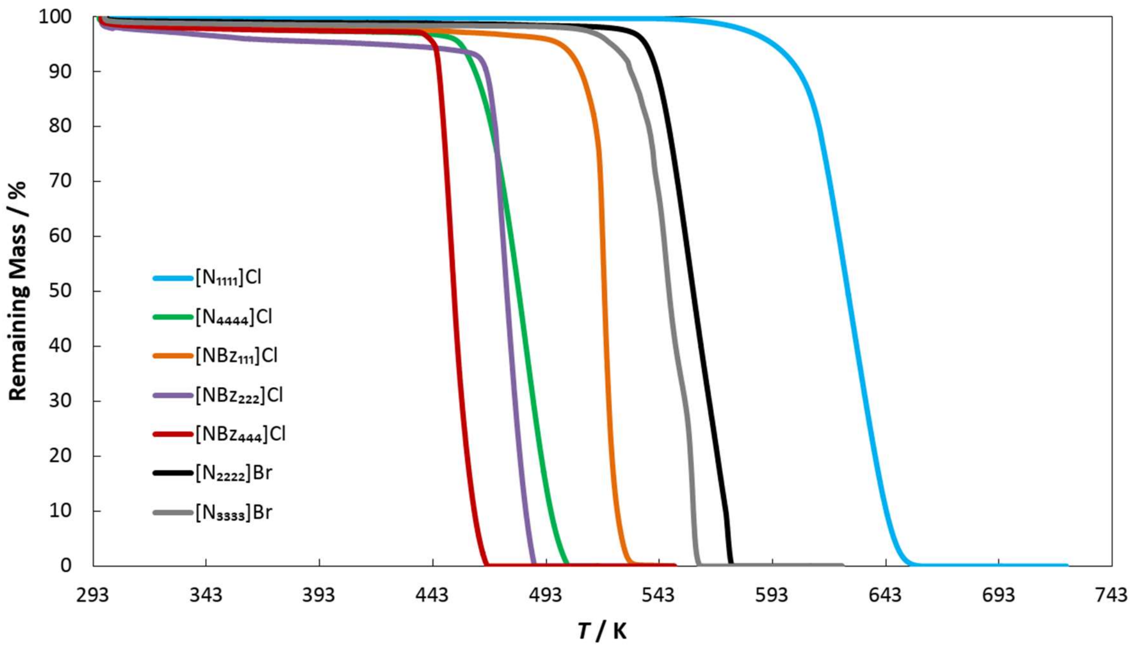

Figure S1. TGA thermograms of the quaternary ammonium salts used in this work.

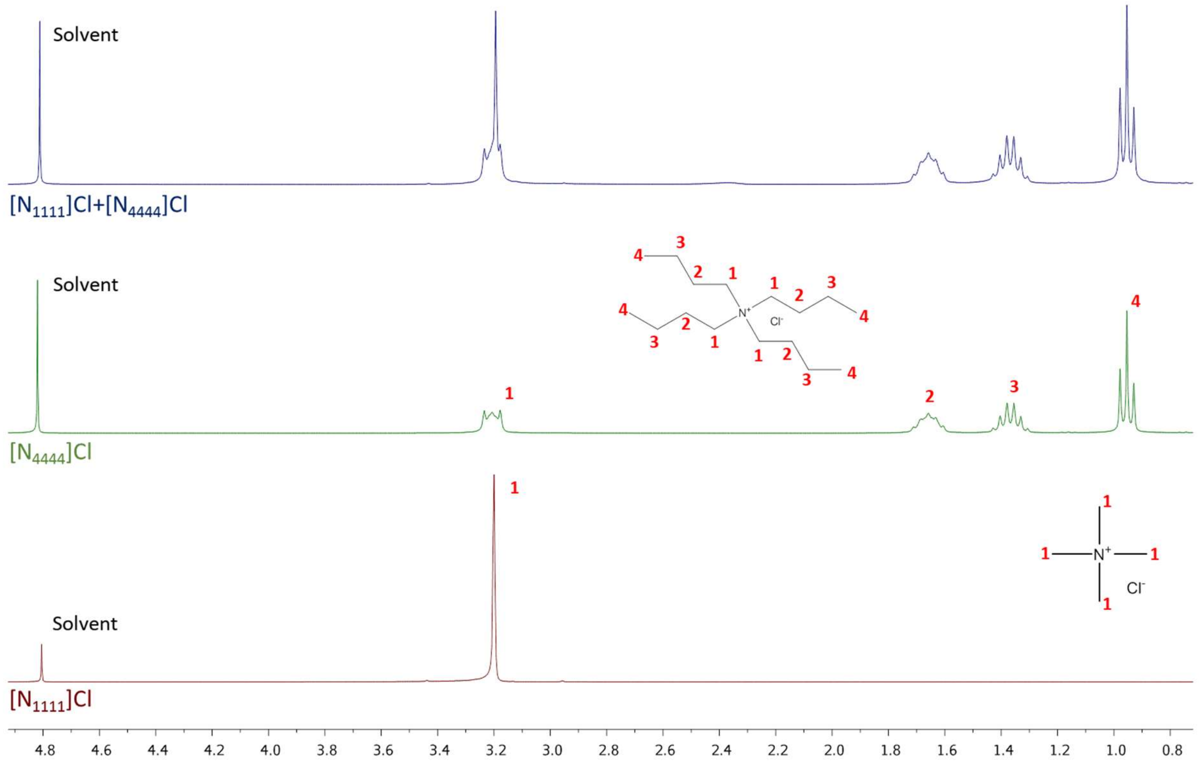

Figure S2. ^{1}H-NMR spectra of $[N_{1,1,1,1}]Cl$, $[N_{4,4,4,4}]Cl$ and the mixture $[N_{1,1,1,1}]Cl+[N_{4,4,4,4}]Cl$ at the eutectic composition (following melting and recrystallisation) in deuterated water as solvent.

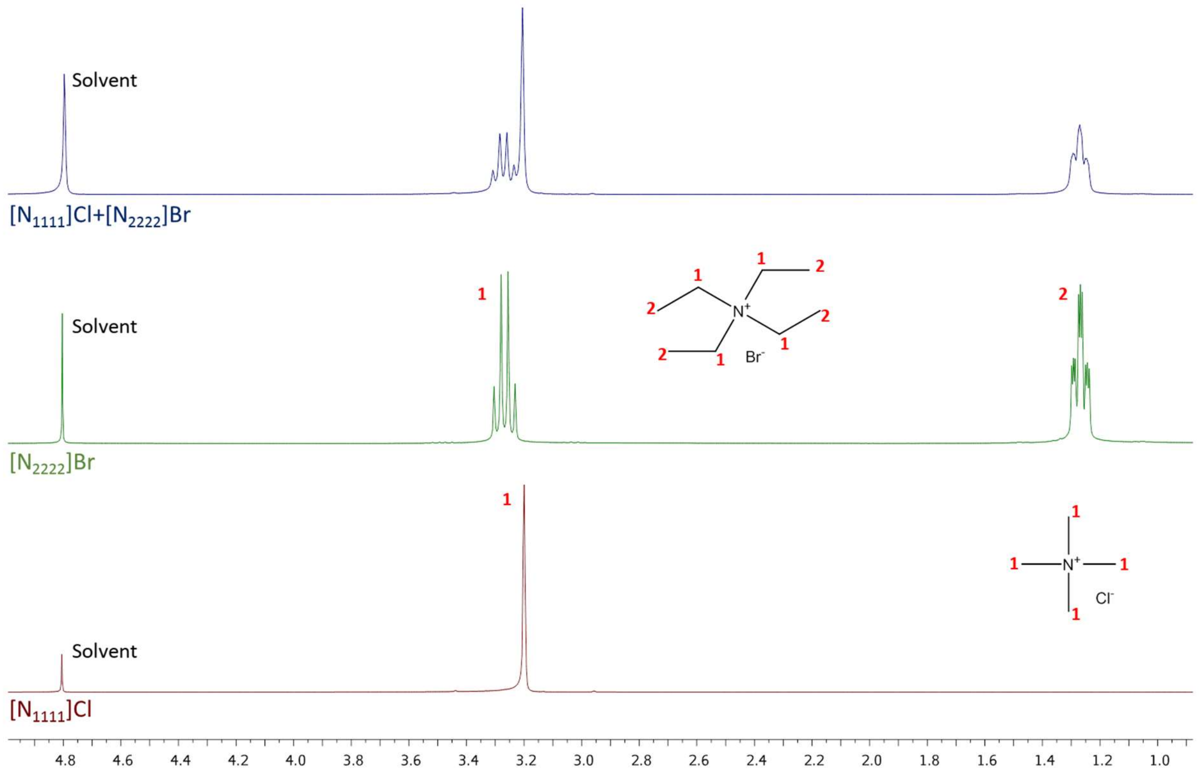

Figure S3. ^{1}H-NMR spectra of $[N_{1,1,1,1}]Cl$, $[N_{2,2,2,2}]Br$ and the mixture $[N_{1,1,1,1}]Cl+[N_{2,2,2,2}]Br$ at the eutectic composition (following melting and recrystallisation) in deuterated water as solvent.

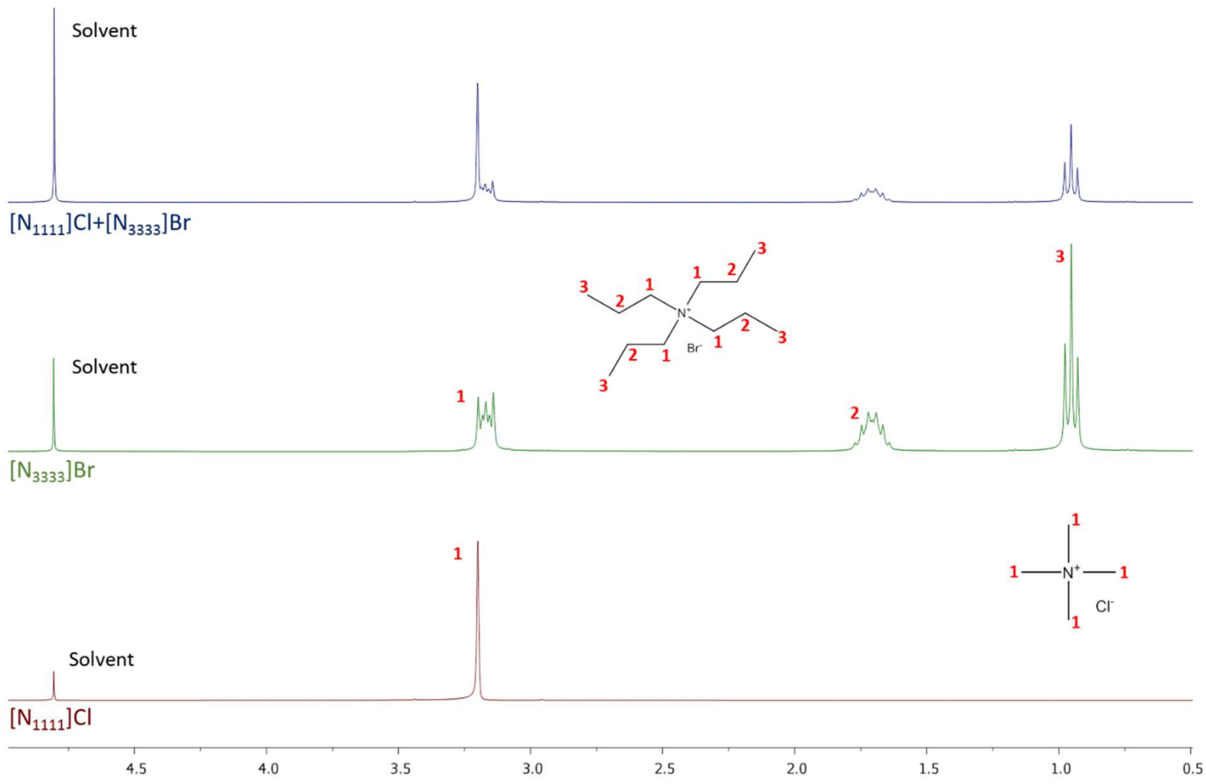

Figure S4. ^{1}H-NMR spectra of [$N_{1,1,1,1}$]Cl, [$N_{3,3,3,3}$]Br and the mixture [$N_{1,1,1,1}$]Cl+[$N_{3,3,3,3}$]Br at the eutectic composition (following melting and recrystallisation) in deuterated water as solvent.

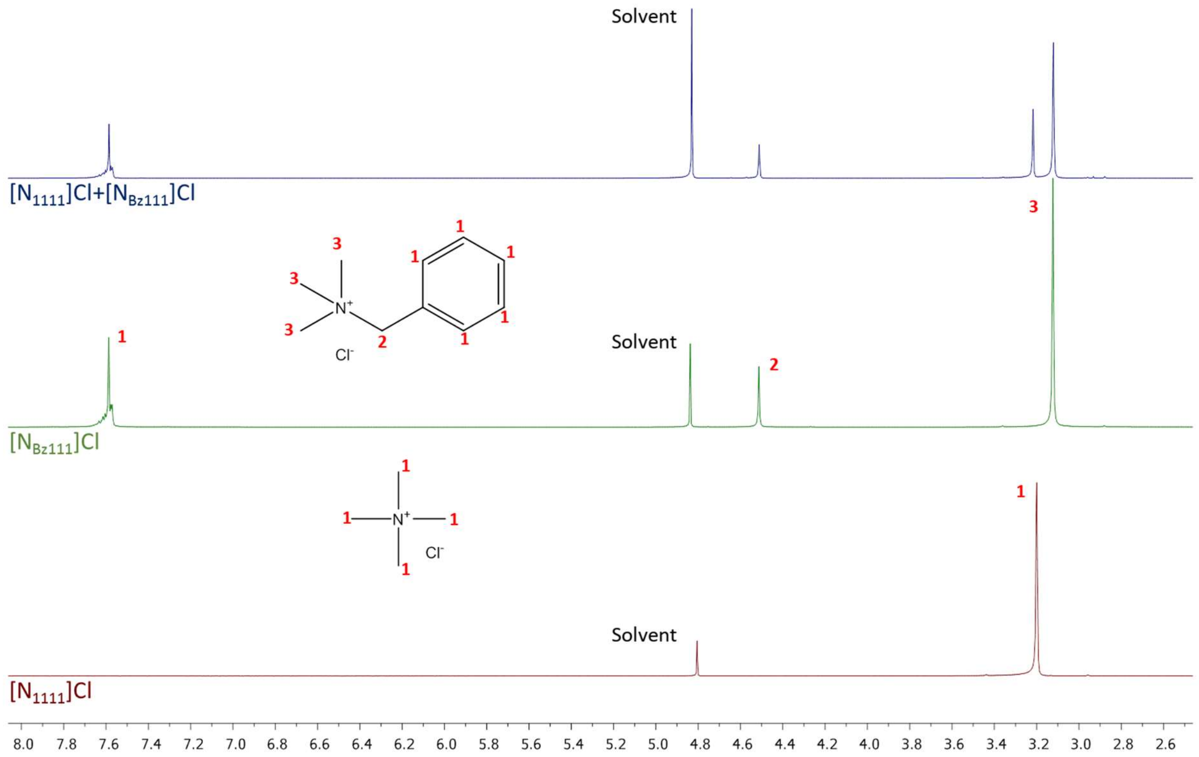

Figure S5. ^{1}H-NMR spectra of $[N_{1,1,1,1}]$Cl, $[N_{Bz,1,1,1}]$Cl and the mixture $[N_{1,1,1,1}]$Cl+$[N_{Bz,1,1,1}]$Cl at the eutectic composition (following melting and recrystallisation) in deuterated water as solvent.

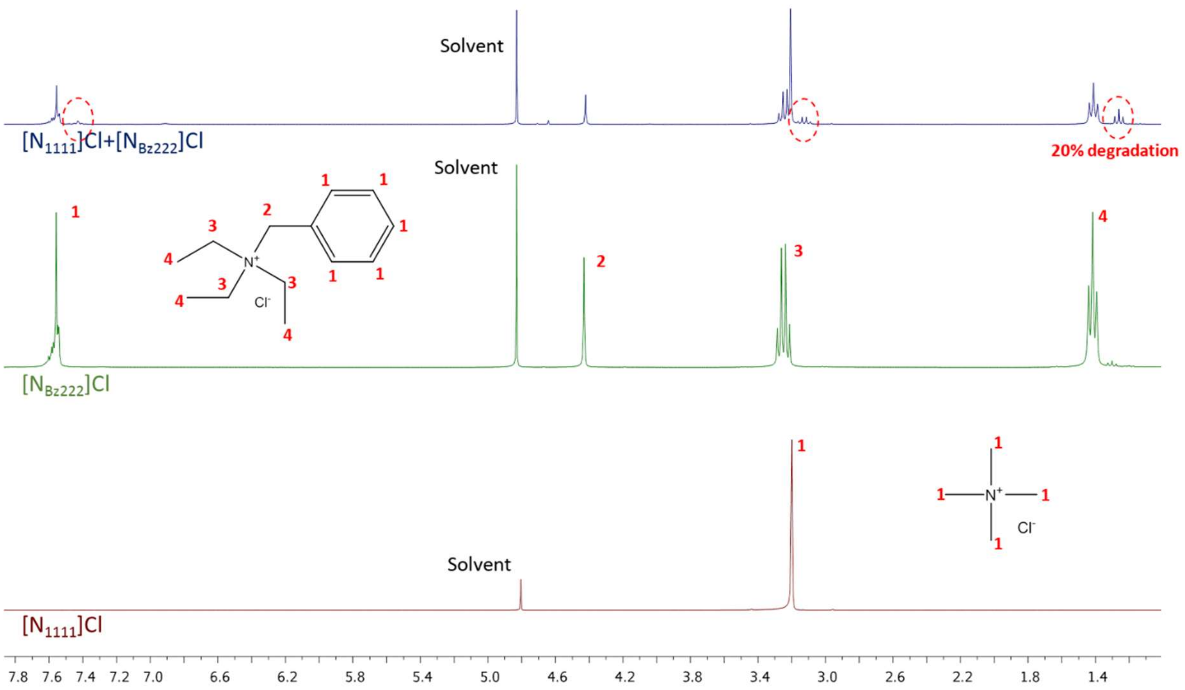

Figure S6. ^{1}H-NMR spectra of $[N_{1,1,1,1}]Cl$, $[N_{Bz,2,2,2}]Cl$ and the mixture $[N_{1,1,1,1}]Cl+[N_{Bz,2,2,2}]Cl$ at the eutectic composition (following melting and recrystallisation) in deuterated water as solvent. Peaks attributed to degradation products are circled in red.

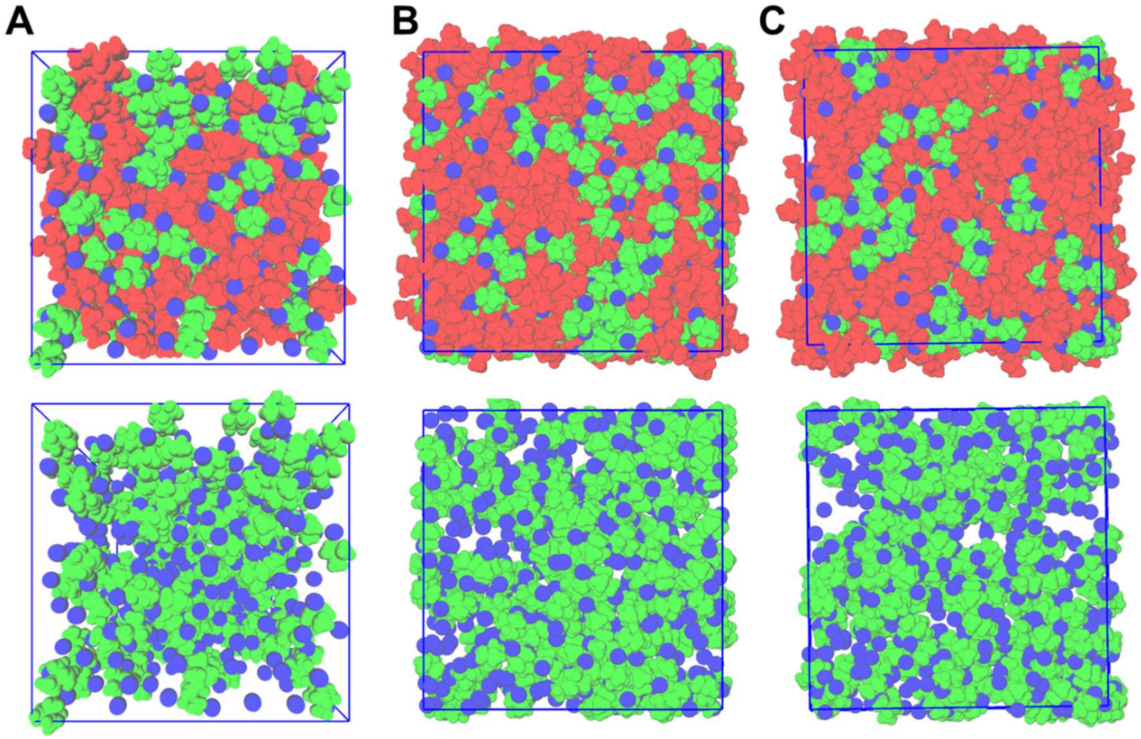

Figure S7. Top: Final simulation snapshot of the liquid-phase equimolar mixture of A) $[N_{1,1,1,1}]Cl+[N_{2,2,2,2}]Cl$ at 533 K, $[N_{1,1,1,1}]Cl+[N_{3,3,3,3}]Cl$ at 423 K and $[N_{1,1,1,1}]Cl+[N_{4,4,4,4}]Cl$ at 353 K. Bottom: Identical system with $[N_{x,x,x,x}]^+$(x=2-4) removed to better identify the presence of $[N_{1,1,1,1}]^+$-Cl^- clusters. Colour scheme: green for $[N_{1,1,1,1}]^+$, red for $[N_{x,x,x,x}]^+$(x=2-4) and blue for Cl^- .

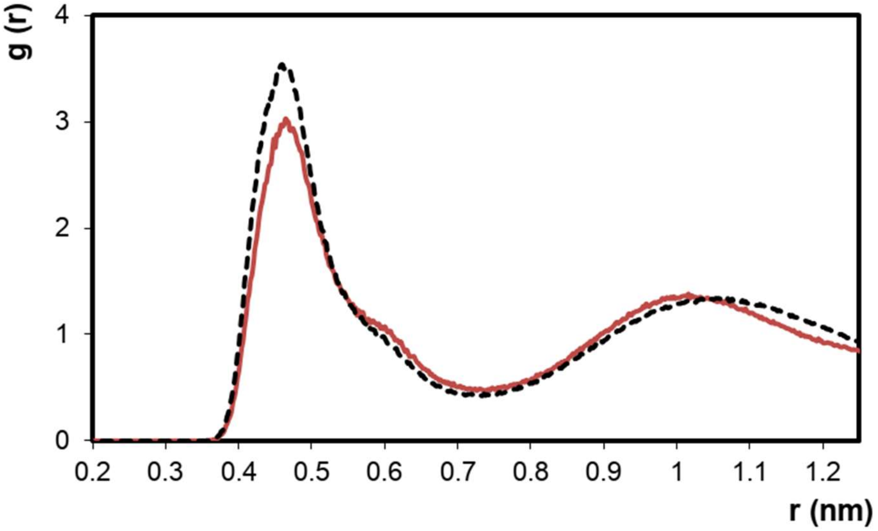

Figure S8. RDFs of chloride around the $[N_{2,2,2,2}]^+$ cation in the corresponding chloride-salt hypothetical pure liquid phase (dashed line) and in the equimolar liquid phase mixtures of

$[N_{1,1,1,1}]Cl+[N_{2,2,2,2}]Cl$ (——) at 533.15 K. In each case, the x-axis represents the distance of the chloride ion from the central nitrogen of the cation.

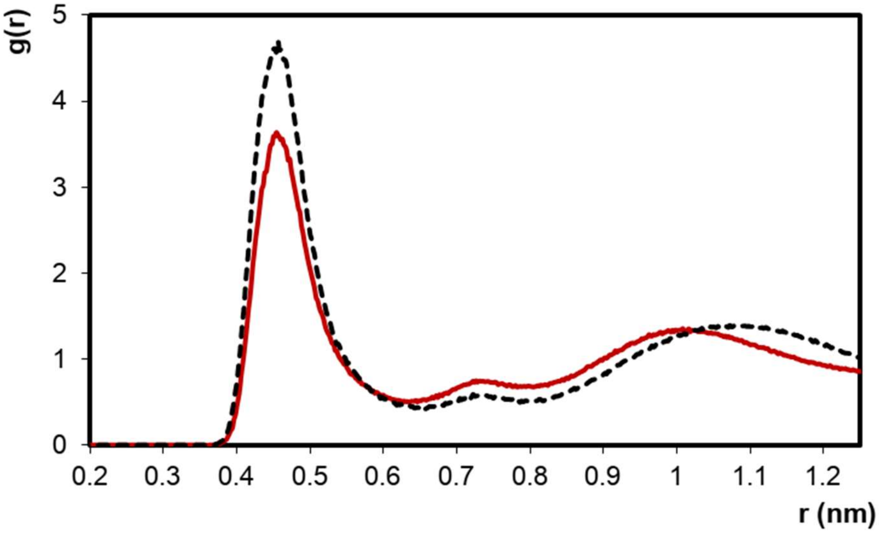

Figure S9. RDFs of chloride around the $[N_{3,3,3,3}]^+$ cation in the corresponding chloride-salt hypothetical pure liquid phase (dashed line) and in the equimolar liquid phase mixtures of $[N_{1,1,1,1}]Cl+[N_{3,3,3,3}]Cl$ (——) at 423.15 K. In each case, the x-axis represents the distance of the chloride ion from the central nitrogen of the cation.

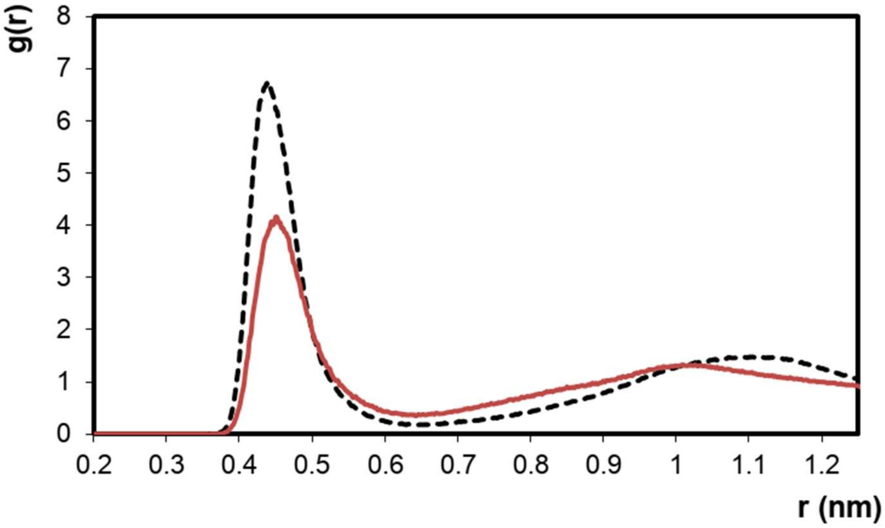

Figure S10. RDFs of chloride around the $[N_{4,4,4,4}]^+$ cation in the corresponding chloride-salt hypothetical pure liquid phase (dashed line) and in the equimolar liquid phase mixtures of $[N_{1,1,1,1}]Cl+[N_{4,4,4,4}]Cl$ (——) at 353.15 K. In each case, the x-axis represents the distance of the chloride ion from the central nitrogen of the cation.
